# Maternal and perinatal outcomes of dengue in PortSudan, Eastern Sudan

**DOI:** 10.1186/1743-422X-7-153

**Published:** 2010-07-13

**Authors:** Ishag Adam, Ammar M Jumaa, Hagir M Elbashir, Mubarak S Karsany

**Affiliations:** 1 Faculty of Medicine, University of Khartoum, Khartoum, Sudan; 2 Faculty of Medicine, Red Sea University, PortSudan, Sudan; 3 Faculty of Medicine, Juba University, Khartoum, Sudan

## Abstract

**Aim:**

To investigate maternal and perinatal outcomes (maternal death, preterm delivery, low birth weight and perinatal mortality) of dengue at PortSudan and Elmawani hospitals in the eastern Sudan.

**Method:**

This was a retrospective Cohort study where medical files of women with dengue were reviewed.

**Results:**

There were 10820 deliveries and 78 (0.7%) pregnant women with confirmed dengue IgM serology at the mean (SD) gestational age of 29.4(8.2) weeks. While the majority of these women had dengue fever (46, 58.9%), hemorrhagic fever and dengue shock syndrome were the presentations in 18 (23.0%) and 12, (15.3%) of these women, respectively. There were 17(21.7%) maternal deaths. Fourteen (17.9%) of these 78 women had preterm deliveries and 19 (24.3%) neonates were admitted to neonatal intensive care unit. Nineteen (24.3%) women gave birth to low birth weight babies. There were seven (8.9%) perinatal deaths. Eight (10.2%) patients delivered by caesarean section due to various obstetrical indications.

**Conclusion:**

Thus dengue has poor maternal and perinatal outcomes in this setting. Preventive measures against dengue should be employed in the region, and more research on dengue during pregnancy is needed.

## Introduction

Dengue is the most common mosquito-borne infection, with an estimated 100 million infections worldwide per year [1-3]. Many factors like urbanization, increased population density, air travel, and limited resources for dengue prevention has led to dengue becoming a major public health problem in the tropics [3]. Of the 100 million annual infections, 250-500 thousand persons manifest severe disease, with the remainder being mild, nonspecific, or even asymptomatic [1-3].

Classic dengue fever (DF) is defined by the World Health Organization as an acute febrile illness with two more of the following signs or symptoms: intense headache, retro-orbital pain, myalgia, arthralgia, rash, leucopenia, and a hemorrhagic manifestation [4]. A small proportion of infected persons develop (dengue hemorrhagic fever (DHF), which is characterized by fever, thrombocytopenia, hemorrhagic manifestations, and increased vascular permeability with plasma leakage primarily into the pleural cavity and peritoneum [5]. The main clinical feature differentiating DF from DHF and dengue shock syndrome (DSS) is the increased vascular permeability, which, if unrecognized or not judiciously treated may result in hypovolemic shock, organ impairment, and death [6].

Dengue during pregnancy may be associated with various complications, including maternal mortality, preterm delivery, fetal death, low birth weight, neonatal admissions, fetal anomalies, and miscarriage [7-14]. However, the vast majority of these reports were case series and from south East Asia. Thus, there is an urgent need to investigate the presentation and outcomes of dengue so as to provide caregivers and health planners with fundamental data necessary for the practicing clinicians as well as researchers.

Dengue have been reported in different regions of the Sudan - which the largest African country - including the study area PortSudan [15,16]. Despite the prevalence of dengue in Sudan, there are no data on the maternal and fetal consequences of dengue during pregnancy. The present study was conducted to investigate maternal and perinatal outcomes of dengue infection at PortSudan and Elmawani maternity hospitals in the eastern Sudan.

## Methods

All dengue cases presented at PortSudan and Elmawani hospitals during 2008 - 2009 were reviewed retrospectively. These two maternity hospitals provide tertiary care for women who receive antenatal care at the hospital, as well as for referrals from other clinics and hospitals, and for women who live close to the hospital facility. All women with risk factors or obstetric complications are referred to this hospital. However, the referral criteria are not strictly adhered to and many patients without any significant complications presented at the hospital.

The medical files of all women with dengue were reviewed; patients' records retrieved and the age, parity, residence, gestational and maternal and perinatal outcomes and biochemical characteristics were recorded. Following our previous collaborations with Sudanese national ministry of health in the different epidemics [17,18] we have been consulted during this dengue epidemic and the diagnosis or exclusion of dengue was conducted in the epidemiological lab in Khartoum under supervision of one of our team (MSK).

A maternal death was defined as the death of a woman while pregnant or within 42 days of termination of pregnancy, irrespective of the duration and site of the pregnancy, from any cause.

Miscarriage was defined as the expulsion of the fetus before 28 weeks of gestation, and preterm delivery as a delivery that occurred between 28 and 37 weeks of pregnancy. Low birth weight is the baby delivered weighting <2500 gm. Perinatal mortality was defined as the number of deaths of newborns born after ≥28 weeks of gestation till the end of day 7.

## Statistics

Data were entered into a computer database and SPSS software (SPSS Inc., Chicago, IL, USA, version 13.0) and double checked before analysis. Values are reported as frequency, percentage, and mean (SD).

## Results

During the period of study there were 10820 deliveries and 78 (0.7%) pregnant women with confirmed dengue serology IgM using ELISA at the mean (SD) gestational age of 29.4(8.2) weeks. While the majority of these cases were dengue fever (46, 58.9%), hemorrhagic fever and dengue shock syndrome were the presentations in (18, 23.0%) and (12, 15.3%), respectively. The socio-demographic characteristics were shown in table 1. Various symptoms were observed among these patients including headache, fever, muscle pain or arthralgia, abdominal pain, retro-orbital pain and rash. Five (6.4%) and three (3.8%) patients had vaginal bleeding and epistaxis, respectively.

**Table 1:** Baseline socio-demographic and biochemical characteristics of the 78 pregnant patients with dengue

Characters	Mean (SD)
Age, years	28.9(6.8)
Parity	2.8(2.3)
Duration of illness	5.6 (2.3)
Weight, kg	62(6.8)
Gestational age	29.4(8.2)
Temperature	37.8 (0.8)
Haemoglobin, gm/dl	7.9(2.7)
Blood glucose, mg/dL	121.5 (8. 9)
Creatinine, mg/dL	1.4 (0.6)
Platelets, cells/μL	128, 568 (18.225)
White blood cells, cells/μL	3425 (4565.7)
Total protein, g/dL	7.8 (0.78)
Serum bilirubin, mg/dL	1.2 ± 0.9
Alanine aminotransferase, IU/L	65.9 ± 21.7
Aspartate aminotransferase, IU/L	82.6 ± 19.5

There were 17(21.7%) maternal death due to multiple organ failure and haemorrhage; all of these deaths were dengue hemorrhagic fever and dengue shock syndrome. Fourteen (17.9%) of these 78 women had preterm deliveries and 19 (24.3%) neonates were admitted to neonatal intensive care unit. Nineteen (24.3%) women gave birth to low birth weight babies. There was no fetal anomaly. There were seven (8.9%) perinatal deaths. Eight (10.2%) patients delivered by caesarean delivery due to various obstetrics indications, table 2.

**Table 2:** Maternal and perinatal outcomes in 78 pregnant patients with dengue

Variable	Number	Percentage
Maternal death	17	21.7
Low birth weight deliveries	19	24.3
Preterm delivery	14	17.9
Cesarean delivery	8	10.2
Admission to neonatal intensive care unit	19	24.3
Perinatal death	7	8.9

## Discussion

Perhaps this is largest study investigated maternal and perinatal outcomes of dengue during pregnancy. The main findings of this study were the poor maternal and perinatal outcomes due to dengue mainly maternal deaths (21.7%) and preterm deliveries (24.3%). We recently reported a high maternal mortality in different regions of Sudan due to various infectious diseases [19,20] for example four (4.9%) out of 42 and 25% of the sixteen women died due to visceral leishmaniasis and viral hepatitis, respectively [17,21]. However, in Sudan, as in many sub-Saharan countries, the frequency rates of miscarriage, preterm labor, and congenital abnormalities in the general population are unknown. In Malaysia, out of 16 patients with dengue, there were three cases of maternal death, 50.0% of the women had preterm deliveries and three babies required intensive care [9]. Previously, it have been reported that maternal clinical dengue may be associated with pregnancy complications, including maternal mortality, preterm delivery, fetal death, low birth weight, neonatal admissions, fetal anomalies, and miscarriage [7-14]. These reports should be compared with ours cautiously, because in this setting we might have observed the severe form of the disease. In contrast to other Asiatic countries, dengue is not endemic in Sudan and epidemics occur from time to another, which explains the lack of immunity in the population and the severity of the disease. Hyperendemic conditions are associated with an increased probability of secondary infections and occurrence of virulent strains. Despite these trends, little research has been conducted to examine the impact of the severity of maternal dengue infection. This is an important factor to assess because viral titers are thought to vary by severity of infection, and pathogenesis associated with poor pregnancy outcomes can be caused by either the direct effect of the virus or the body's response to high titers [22-24]. On the other hand there were no any significant differences in pregnancy outcome comparing dengue IgM-positive with IgM-negative women, perhaps in mild or asymptomatic dengue in pregnant women [25]. However, our study was a hospital-based one which might not reflect what was at the community level.

In the current study, 5 (6.4%) patients had vaginal bleeding and 8 (10.2%) patients delivered by caesarean delivery. The risk of maternal haemorrhage was reported before when caesarean section was carried out on patient with dengue fever [14]. Furthermore, difficulties in maintaining haemostasis during the caesarean section were observed too [14]. Deliveries were sometimes pathological, so the risk of haemorrhage has to be assessed and precautions should be taken both for natural delivery and caesarean section. Generally in tropics the situations sometimes are not clear and many infections/toxicity may mimic the obstetrical emergency for example we recently observed association between antepartum haemorrhage and snake bite [26].The impact of dengue on pregnancy is not fully understood. Studies in the literature are limited and case studies were too small to draw conclusions.

## Conclusion

Thus dengue has poor maternal and perinatal outcomes in this setting. Preventive measures against dengue should be employed in the region, and more research on dengue during pregnancy is needed.

## Conflicts of interest statement

The authors declare that they have no competing interests.

## Authors' contributions

IA, HME and MSK designed the study; AM, HME and IA carried out the clinical work and analysis and interpretation of these data. All authors read and approved the final manuscript.

## Funding

The study was funded by Pharma Xir, Khartoum, Sudan.

## Ethics

The study received ethical clearance from the Research Board at the Faculty of Medicine, University of Khartoum, Sudan.
